# Co-Morbid Obsessive–Compulsive Disorder and Attention Deficit Hyperactivity Disorder: Neurobiological Commonalities and Treatment Implications

**DOI:** 10.3389/fpsyt.2019.00557

**Published:** 2019-08-13

**Authors:** Sonja Cabarkapa, Joel A. King, Nathan Dowling, Chee H. Ng

**Affiliations:** ^1^The Melbourne Clinic, Richmond, VIC, Australia; ^2^St Vincent’s Health, Melbourne, VIC, Australia; ^3^The University of Melbourne, Melbourne, VIC, Australia

**Keywords:** OCD, ADHD (attention deficit and hyperactivity disorder), co-morbid, neurobiology, treatment

## The Impact of Co-Morbid OCD–ADHD

The prevalence and functional impairment for OCD–ADHD appears substantial. A recent study has shown a prevalence rate of 11.8% for co-morbid ADHD in OCD-affected individuals (1). Pediatric OCD patients showed a prevalence estimate of 25.5% for co-morbid ADHD (2). Co-morbid OCD–ADHD from a young age was associated with greater OCD severity and persistence of symptoms in a prospective follow-up period (3), and poorer prognosis (4). Studies suggest that patients experience more disabling OCD symptoms when ADHD is also present, but the actual nature of OCD symptoms are not significantly different from patients with OCD alone (5, 6). The varying prevalence of co-morbidity is most likely due to differences in study definitions and methodology, but raises questions on whether both these disorders are actually present simultaneously, or whether one disorder may mimic or predispose an individual to the other disorder (7). Their co-morbidity also behoves clinicians to consider undiagnosed ADHD in treatment-resistant OCD, and vice versa.

Previously, ADHD and OCD were viewed very distinctly on an impulsive–compulsive continuum. However, there has been increasing awareness of the cross-cutting nature of the symptoms domains of impulsivity and compulsivity (8). Impulsivity is characterized by inability to resist impulses and urges, delaying gratification deficits, unreflective decision making, and premature behaviour. Compulsivity is characterized by perseverative and repetitive action, and is ruminative and rigid in nature. Both these constructs co-occur in separate cases of ADHD and OCD at varying severities. Patients with OCD can feel urges to enact compulsions when prevented from doing so, and may suddenly yield to them, despite full awareness of the negative consequences. Patients with ADHD may chance upon an immediately pleasurable activity that they then repeat for ongoing pleasurable effect.

There is overlap between the two conditions with features such as impulsivity observed in both groups but at markedly higher levels in those with ADHD (9).

## Neurobiology of ADHD and OCD

**Figure 1:**
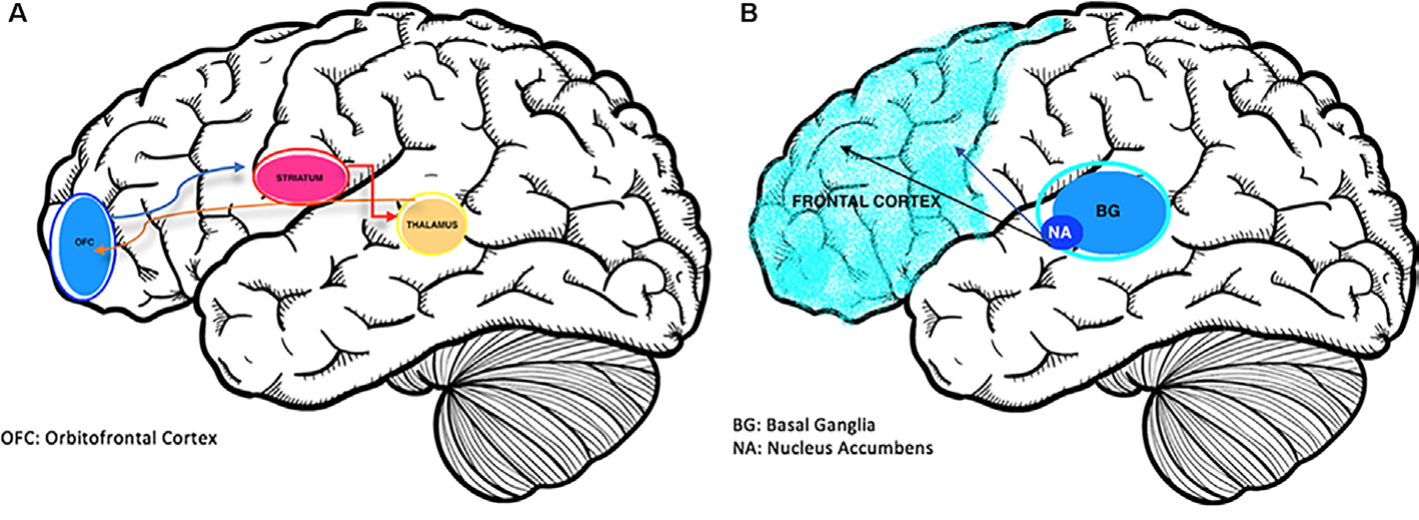
**(A)** The Neurobiology of OCD (12); overactivation of the cortico-striatal-thalamic-cortical loop is associated with obsessions. **(B)** Brain changes in ADHD (13); reduced activation of prefrontal and striatal regions including the nucleus accumbens and basal ganglia structures.

Frontostriatal pathophysiology appears to be involved in both disorders (see **Figures 1A, B**). Brain-mapping studies have shown that patients with ADHD and OCD have shared but also disorder-specific brain dysfunctions during interference inhibition and attention allocation suggesting to be the result of alternate dopamine modulation of striatal brain regions (10). Predominantly, the overlap in activation deficits in ADHD and OCD are in frontostriato-insular-cerebellar regions. These regions play a role in self-control and temporal foresight, and govern impulsivity with choices (11).

While ADHD is characterized with frontostriatal hypoactivity (14), OCD commonly appears hyperactive in this region on functional imaging. Patients with OCD alone showed abnormalities in functioning of the right orbitofrontal and rostrolateral prefrontal cortex (15). Conversely, the disinhibition and impulsivity of ADHD are associated with hypoperfusion of prefrontal regions (10).

There are also disorder-specific structural abnormalities. Patients with OCD tend to have larger and hyperfunctioning insular-striatal regions, whereas those with ADHD have smaller and underfunctioning ventrolateral prefrontal/insular-striatal regions (16).

The degree of frontostriatal hyperactivity or hypoactivity also appears to underpin symptoms in both OCD and ADHD. In ADHD, there is an inverse correlation between impulsive symptoms with activity in prefrontal (17, 18) and striatal (19) regions. Recent findings support this by highlighting an inverse correlation between frontostriatal functional connectivity and symptom severity in ADHD (14, 20). Alternately, OCD demonstrates a positive association between symptom severity and brain activity (21–24). A recent study comparing OCD and ADHD patients identified that the correlation of symptom severity with frontostriatal brain activity was positive in the OCD sample but negative in the ADHD sample (9). These contrasting findings may support the hypothesis that these disorders lie on opposite ends of a clinical impulsive–compulsive continuum.

However, despite these differences, OCD and ADHD appear to share similar neuropsychological impairments in executive functions, particularly inhibition deficit (25). This suggests that different neurobiological processes can lead to similar dysfunction (25). When using learned reward expectancies to guide decision making, findings suggest an overlap in deficits, as well as shared dysfunction in medio-fronto-striato-limbic brain regions. However, results also suggest the existence of unique dysfunction in the ventromedial orbitofrontal cortex in OCD, and in the right putamen in ADHD, indicating additional disorder-specific abnormalities (26).

## Treatment Implications for Co-Morbid OCD–ADHD

Based on current neurobiological knowledge, OCD and ADHD appear to have different and apparently opposing deficits in the frontostriatal and related areas. This may have treatment implications in co-morbid OCD–ADHD.

The effectiveness of selective serotonin reuptake inhibitors (SSRIs) in the treatment of OCD has been well established (27). These agents regulate hyperactivity in the frontostriatal region of the brain (28, 29). In cases of OCD patients who exhibit only partial response to SSRIs, antipsychotics with dopamine blocking properties appear to have a useful effect in augmenting serotonin reuptake inhibition (30).

On the other hand, first line treatment for ADHD has been methylphenidate or dexamphetamine for several decades. Stimulant medication increases prefrontal activation and significantly improves both clinical symptomology and neurocognitive processes through modulation of dopamine reuptake. In addition, ADHD-tailored CBT which address academic and organizational skills training, problem-solving and prioritizing tasks, as well as managing unhelpful thinking and behavioral patterns, demonstrate encouraging improvements in adolescents and adults (31, 32).

Some literature suggests that stimulants can exacerbate and provoke OCD symptoms. While this may be consistent with the theoretical understanding of dopaminergic prefrontal hyperactivity, this evidence is mostly anecdotal and remains limited (33–37).

In terms of co-morbid OCD–ADHD, there is evidence to support that treating OCD with SSRIs and cognitive behaviour therapy with exposure response prevention (CBT+ERP) improves attentional symptoms (38). This may be mediated through SSRI activation of BDNF and other neurotrophic factors that lead to neuronal growth in networks responsible for working memory and processing speed (39–41).

There is also evidence that treating ADHD with stimulants improves co-morbid obsessive–compulsive symptoms. CBT+ERP has demonstrated efficacy for children and adults with OCD, but untreated co-morbid ADHD diminishes treatment response on the OCD (7). Stimulant treatment improves attention, conscious learning and retention of CBT skills and allows patients to apply skills when obsessive thoughts recur.

Limiting treatment to only one disorder when both are present appears to be associated with poorer outcomes. A study investigating the use of paroxetine in patients with co-morbid ADHD found that the OCD response rates were significantly less than in patients with OCD alone. The presence of ADHD co-morbidity was also associated with a greater rate of OCD relapse (42). Conversely, case studies suggest that treating both disorders concurrently can be beneficial for both disorders (43).

It would therefore seem prudent to treat both conditions concurrently when they are both present and severe enough to warrant biological and psychological treatments. Additionally, screening for disorders of addiction is important due to the heightened cumulative risk in these individuals. Despite the lack of randomized controlled trials in clinical populations, it would be reasonable to start with standard treatments for both, such as SSRIs for OCD and psychostimulants such as methylphenidate and dexamphetamine for ADHD. Pharmacological treatments should be introduced one at a time to avoid confusion around treatment response and side-effects. This is especially relevant given the possibility of worsening OCD with stimulants. Monitoring progress through routine use of established instruments such as the Yale Brown Obsessive Compulsive Scale for OCD (44) and ADHD Rating Scale 5 or similar (45) is appropriate.

Transcranial magnetic stimulation (TMS) has a limited but growing body of evidence for its efficacy in both ADHD (46–48) and OCD (49, 50) Combined with its relative safety, TMS may be a viable option for treating co-morbid ADHD and OCD, pending further clinical trials. Additionally, where symptoms remain resistant to both pharmacological and psychological approaches and OCD symptoms significantly disrupt daily functioning, intensive residential treatment may be considered if available (51).

## Conclusion

The treatment of OCD–ADHD co-morbidity in children, and to an even greater extent in adults, remains challenging. ADHD and OCD are not mutually exclusive and do co-exist (52). They share dysfunction in impulsivity, inattention, and executive function while appearing to have opposing pathophysiology and phenomenology. Treating one without the other leads to poorer outcomes in both, while treating both simultaneously is associated with better outcomes. Further research is needed to better understand the neurobiological interplay between both disorders, refine our nosology of them, and guide treatment options.

## Author Contributions

JK, SC, and ND contributed to the conception of the paper. SC wrote the first draft of the manuscript, with contributions from ND. JK significantly revised the manuscript into its near completed form. CN provided final corrections and additions. All authors read and approved the submitted version.

## Conflict of Interest Statement

JK has received speaker honorarium from Servier. CN had served as a consultant for Lundbeck, Grunbiotics, Servier, Janssen-Cilag, Wyeth and Eli Lilly, received research grant support from Wyeth and Lundbeck, and speaker honoraria from Servier, Lundbeck, Bristol-Myers Squibb, Organon, Eli Lilly, GlaxoSmithKline, Janssen- Cilag, Astra-Zenaca, Wyeth, and Pfizer.

The remaining authors declare that the research was conducted in the absence of any commercial or financial relationships that could be construed as a potential conflict of interest.

## References

[1] Sheppard B Chavira D Azzam A Grados MA Umaña P Garrido H ADHD prevalence and association with hoarding behaviors in childhood-onset OCD. Depress Anxiety (2010) 27(7):667–74. 10.1002/da.20691 PMC2925836 20583294

[2] Masi G Millepiedi S Perugi G Pfanner C Berloffa S Pari C A naturalistic exploratory study of the impact of demographic, phenotypic and comorbid features in pediatric obsessive–compulsive disorder. Psychopathology (2010) 43(2):69–78. 10.1159/000274175 20068377

[3] Walitza S Zellmann H Irblich B Lange KW Tucha O Hemminger U Children and adolescents with obsessive–compulsive disorder and comorbid attention-deficit/hyperactivity disorder: preliminary results of a prospective follow-up study. J Neural Transm (2008) 115(2):187–90. 10.1007/s00702-007-0841-2 18200431

[4] Grados M Riddle MA Do all obsessive–compulsive disorder subtypes respond to medication? Int Rev Psychiatry (2008) 20(2):189–93. 10.1080/09540260801889153 18386211

[5] Masi G Millepiedi S Mucci M Bertini N Pfanner C Arcangeli F Comorbidity of obsessive–compulsive disorder and attention-deficit/hyperactivity disorder in referred children and adolescents. Compr Psychiatry (2006) 47(1):42–7. 10.1016/j.comppsych.2005.04.008 16324901

[6] Geller DA Coffey B Faraone S Hagermoser L Zaman NK Farrell CL Does comorbid attention-deficit/hyperactivity disorder impact the clinical expression of pediatric obsessive–compulsive disorder? CNS Spectr (2003) 8(4):259–64. 10.1017/S1092852900018472 12679741

[7] Geller DA Biederman J Faraone SV Cradock K Hagermoser L Zaman N Attention-deficit/hyperactivity disorder in children and adolescents with obsessive–compulsive disorder: fact or artifact? J Am Acad Child Adolesc Psychiatry (2002) 41(1):52–8. 10.1097/00004583-200201000-00011 11800207

[8] Berlin GS Hollander E Compulsivity, impulsivity, and the DSM-5 process. CNS Spectr (2014) 19:62–8. 10.1017/S1092852913000722 24229702

[9] Abramovitch A Dar R Mittelman A Wilhelm S Comorbidity between attention deficit/hyperactivity disorder and obsessive–compulsive disorder across the lifespan: a systematic and critical review. Harv Rev Psychiatry (2015) 23(4):245–62. 10.1097/HRP.0000000000000050 PMC4495876 26052877

[10] Rubia K Cubillo A Woolley J Brammer MJ Smith A Disorder-specific dysfunctions in patients with attention-deficit/hyperactivity disorder compared to patients with obsessive-compulsive disorder during interference inhibition and attention allocation. Hum Brain Mapp (2011) 32(4):601–11. 10.1002/hbm.21048 PMC6870444 21391250

[11] Norman LJ Carlisi CO Christakou A Cubillo A Murphy CM Chantiluke K Shared and disorder-specific task-positive and default mode network dysfunctions during sustained attention in paediatric attention-deficit/hyperactivity disorder and obsessive/compulsive disorder. Neuroimage (2017) 15:181–93. 10.1016/j.nicl.2017.04.013 PMC5429245 28529874

[12] Stahl SM Stahl SM Stahl’s essential psychopharmacology: neuroscientific basis and practical applications. New York, USA: Cambridge University Press (2013).

[13] Tripp G Wickens JR Neurobiology of ADHD. Neuropharmacology (2009) 57(7–8):579–89. 10.1016/j.neuropharm.2009.07.026 19627998

[14] Konrad K Eickhoff SB Is the ADHD brain wired differently? A review on structural and functional connectivity in attention deficit hyperactivity disorder. Hum Brain Mapp (2010) 31(6):904–16. 10.1002/hbm.21058 PMC6871159 20496381

[15] Norman LJ Carlisi CO Christakou A Chantiluke K Murphy C Simmons A Neural dysfunction during temporal discounting in paediatric attention-deficit/hyperactivity disorder and obsessive–compulsive disorder. Psychiatry Res Neuroimaging (2017) 269:97–105. 10.1016/j.pscychresns.2017.09.008 28988149 PMC5647646

[16] Norman LJ Carlisi C Lukito S Hart H Mataix-Cols D Radua J Structural and functional brain abnormalities in attention-deficit/hyperactivity disorder and obsessive–compulsive disorder: a comparative meta-analysis. JAMA Psychiatry (2016) 73(8):815–25. 10.1001/jamapsychiatry.2016.0700 27276220

[17] Zametkin AJ Liebenauer LL Fitzgerald GA King AC Minkunas DV Herscovitch P Brain metabolism in teenagers with attention-deficit hyperactivity disorder. Arch Gen Psychiatry (1993) 50(5):333–40. 10.1001/archpsyc.1993.01820170011002 8489322

[18] Zametkin AJ Nordahl TE Gross M King AC Semple WE Rumsey J Cerebral glucose metabolism in adults with hyperactivity of childhood onset. N Engl J Med (1990) 323(20):1361–6. 10.1056/NEJM199011153232001 2233902

[19] Cubillo A Halari R Ecker C Giampietro V Taylor E Rubia K Reduced activation and inter-regional functional connectivity of fronto-striatal networks in adults with childhood attention-deficit hyperactivity disorder (ADHD) and persisting symptoms during tasks of motor inhibition and cognitive switching. J Psychiatr Res (2010) 44(10):629–39. 10.1016/j.jpsychires.2009.11.016 20060129

[20] Wolf RC Plichta MM Sambataro F Fallgatter AJ Jacob C Lesch KP Regional brain activation changes and abnormal functional connectivity of the ventrolateral prefrontal cortex during working memory processing in adults with attention-deficit/hyperactivity disorder. Hum Brain Mapp (2009) 30(7):2252–66. 10.1002/hbm.20665 PMC6870879 19107748

[21] Pujol J Soriano-Mas C Alonso P Cardoner N Menchon JM Deus J Mapping structural brain alterations in obsessive–compulsive disorder. Arch Gen Psychiatry (2004) 61(7):720–30. 10.1001/archpsyc.61.7.720 15237084

[22] Lacerda AL Dalgalarrondo P Caetano D Haas GL Camargo EE Keshavan MS Neuropsychological performance and regional cerebral blood flow in obsessive–compulsive disorder. Prog Neuropsychopharmacol Biol Psychiatry (2003) 27(4):657–65. 10.1016/S0278-5846(03)00076-9 12787854

[23] Maltby N Tolin DF Worhunsky P O’Keefe TM Kiehl KA Dysfunctional action monitoring hyperactivates frontal-striatal circuits in obsessive–compulsive disorder: an event-related fMRI study. Neuroimage (2005) 24(2):495–503. 10.1016/j.neuroimage.2004.08.041 15627591

[24] Mataix-Cols D Wooderson S Lawrence N Brammer MJ Speckens A Phillips ML Distinct neural correlates of washing, checking, and hoarding symptom dimensions in obsessive–compulsive disorder. Arch Gen Psychiatry (2004) 61(6):564–76. 10.1001/archpsyc.61.6.564 15184236

[25] Abramovitch A Dar R Hermesh H Schweiger A Comparative neuropsychology of adult obsessive–compulsive disorder and attention deficit/hyperactivity disorder: implications for a novel executive overload model of OCD. J Neuropsychol (2012) 6(2):161–91. 10.1111/j.1748-6653.2011.02021.x 22257360

[26] Norman LJ Carlisi CO Christakou A Murphy CM Chantiluke K Giampietro V Frontostriatal Dysfunction During Decision Making in Attention-Deficit/Hyperactivity Disorder and Obsessive–Compulsive Disorder. Biol Psychiatry Cogn Neurosci Neuroimaging (2018) 3(8):694–703. 10.1016/j.bpsc.2018.03.009 29706587 PMC6278892

[27] Fineberg NA Reghunandanan S Brown A Pampaloni I Pharmacotherapy of obsessive–compulsive disorder: evidence-based treatment and beyond. Aust N Z J Psychiatry (2012) 47(2):121–41. 10.1177/0004867412461958 23125399

[28] Bandelow B Zohar J Hollander E Kasper S Moller HJ Zohar J World federation of societies of biological psychiatry (WFSBP) guidelines for the pharmacological treatment of anxiety, obsessive–compulsive and post-traumatic stress disorders - first revision. World J Biol Psychiatry (2008) 9(4):248–312. 10.1080/15622970802465807 18949648

[29] Pittenger C Kelmendi B Bloch M Krystal JH Coric V Clinical treatment of obsessive compulsive disorder. Psychiatry (Edgmont) (2005) 2(11):34–43. PMC2993523 21120095

[30] Keuneman RJ Pokos V Weerasundera R Castle DJ Antipsychotic treatment in obsessive–compulsive disorder: a literature review. Aust N Z J Psychiatry (2005) 39(5):336–43. 10.1080/j.1440-1614.2005.01591.x 15860020

[31] Chan E Fogler JM Hammerness PG Treatment of attention-deficit/hyperactivity disorder in adolescents: a systematic review. JAMA (2016) 315(18):1997–2008. 10.1001/jama.2016.5453 27163988

[32] Jensen CM Amdisen BL Jørgensen KJ Arnfred SM Cognitive behavioural therapy for ADHD in adults: systematic review and meta-analyses. Atten Defic Hyperact Disord (2016) 8(1):3–11. 10.1007/s12402-016-0188-3 26801998

[33] Koizumi HM Obsessive–compulsive symptoms following stimulants. Biol Psychiatry (1985) 20(12):1332–3. 10.1016/0006-3223(85)90120-9 4063424

[34] Kouris S Methylphenidate-induced obsessive–compulsiveness. J Am Acad Child Adolesc Psychiatry (1998) 37(2):135. 10.1097/00004583-199802000-00001 9473905

[35] Serby M Methylphenidate-induced obsessive–compulsive symptoms in an elderly man. CNS Spectr (2003) 8(8):612–3. 10.1017/S1092852900018885 12907924

[36] Woolley JB Heyman I Dexamphetamine for obsessive–compulsive disorder. Am J Psychiatry (2003) 160(1):183. 10.1176/appi.ajp.160.1.183 12505824

[37] Jhanda S Singla N Grover S Methylphenidate-induced obsessive–compulsive symptoms: a case report and review of literature. J Pediatr Neurosci (2016) 11(4):316–8. 10.4103/1817-1745.199461 PMC5314844 28217153

[38] Guzick AG McNamara JPH Reid AM Balkhi AM Storch EA Murphy TK The link between ADHD-like inattention and obsessions and compulsions during treatment of youth with OCD. J Obsessive Compuls Relat Disord (2017) 12:1–8. 10.1016/j.jocrd.2016.11.004 28966908 PMC5619255

[39] Hall D Dhilla A Charalambous A Gogos JA Karayiorgou M Sequence variants of the brain-derived neurotrophic factor (BDNF) gene are strongly associated with obsessive–compulsive disorder. Am J Hum Genet (2003) 73(2):370–6. 10.1086/377003 PMC1180373 12836135

[40] Cunha C Brambilla R Thomas KL A simple role for BDNF in learning and memory? Front Mol Neurosci (2010) 3:1. 10.3389/neuro.02.001.2010 20162032 PMC2821174

[41] Björkholm C Monteggia LM BDNF — a key transducer of antidepressant effects. Neuropharmacology (2015) 102:72–9. 10.1016/j.neuropharm.2015.10.034 PMC4763983 26519901

[42] Masi G Millepiedi S Perugi G Pfanner C Berloffa S Pari C Pharmacotherapy in paediatric obsessive–compulsive disorder: a naturalistic, retrospective study. CNS Drugs (2009) 23(3):241–52. 10.2165/00023210-200923030-00005 19320532

[43] King J Dowling N Leow F Methylphenidate in the treatment of an adolescent female with obsessive–compulsive disorder and attention deficit hyperactivity disorder: a case report. Australas Psychiatry (2016) 25(2):178–80. 10.1177/1039856216671664 27683657

[44] Goodman WK Price LH Rasmussen SA Mazure C Fleischmann RL Hill CL The Yale-Brown obsessive compulsive scale. I. development, use, and reliability. Arch Gen Psychiatry (1989) 46(11):1006–11. 10.1001/archpsyc.1989.01810110048007 2684084

[45] Ramsay JR Assessment and monitoring of treatment response in adult ADHD patients: current perspectives. Neuropsychiatr Dis Treat (2017) 13:221–32. 10.2147/NDT.S104706 PMC5291336 28184164

[46] Rubio B Boes AD Laganiere S Rotenberg A Jeurissen D Pascual-Leone A Noninvasive brain stimulation in pediatric attention-deficit hyperactivity disorder (ADHD): a review. J Child Neurol (2015) 31(6):784–96. 10.1177/0883073815615672 PMC4833526 26661481

[47] Rubia K Cognitive neuroscience of attention deficit hyperactivity disorder (ADHD) and its clinical translation. Front Hum Neurosci (2018) 12:100. 10.3389/fnhum.2018.00100 29651240 PMC5884954

[48] Paz Y Friedwald K Levkovitz Y Zangen A Alyagon U Nitzan U Deep rTMS for ADHD. Brain Stimul (2017) 10(2):413. 10.1016/j.brs.2017.01.224

[49] Cocchi L Zalesky A Nott Z Whybird G Fitzgerald PB Breakspear M Transcranial magnetic stimulation in obsessive–compulsive disorder: a focus on network mechanisms and state dependence. Neuroimage Clin (2018) 19:661–74. 10.1016/j.nicl.2018.05.029 PMC6047114 30023172

[50] Carmi L Alyagon U Barnea-Ygael N Zohar J Dar R Zangen A Clinical and electrophysiological outcomes of deep TMS over the medial prefrontal and anterior cingulate cortices in OCD patients. Brain Stimul (2018) 11(1):158–65. 10.1016/j.brs.2017.09.004 28927961

[51] Dowling N Thomas N Blair-West S Bousman C Yap K Smith DJ Intensive residential treatment for obsessive–compulsive disorder: outcomes and predictors of patient adherence to cognitive-behavioural therapy. J Obsessive Compuls Relat Disord (2016) 9:82–9. 10.1016/j.jocrd.2016.04.006

[52] Taurines R Schmitt J Renner T Conner AC Warnke A Romanos M Developmental comorbidity in attention-deficit/hyperactivity disorder. Defic Hyperact Disord (2010) 2(4):267–89. 10.1007/s12402-010-0040-0 21432612

